# Modeling electronic health record data using an end-to-end knowledge-graph-informed topic model

**DOI:** 10.1038/s41598-022-22956-w

**Published:** 2022-10-25

**Authors:** Yuesong Zou, Ahmad Pesaranghader, Ziyang Song, Aman Verma, David L. Buckeridge, Yue Li

**Affiliations:** 1 School of Computer Science, McGill University, Montreal, Canada; 2 School of Population and Global Health, McGill University, Montreal, Canada

**Keywords:** Data integration, Computational science, Data mining

## Abstract

The rapid growth of electronic health record (EHR) datasets opens up promising opportunities to understand human diseases in a systematic way. However, effective extraction of clinical knowledge from EHR data has been hindered by the sparse and noisy information. We present Graph ATtention-Embedded Topic Model (GAT-ETM), an end-to-end taxonomy-knowledge-graph-based multimodal embedded topic model. GAT-ETM distills latent disease topics from EHR data by learning the embedding from a constructed medical knowledge graph. We applied GAT-ETM to a large-scale EHR dataset consisting of over 1 million patients. We evaluated its performance based on topic quality, drug imputation, and disease diagnosis prediction. GAT-ETM demonstrated superior performance over the alternative methods on all tasks. Moreover, GAT-ETM learned clinically meaningful graph-informed embedding of the EHR codes and discovered interpretable and accurate patient representations for patient stratification and drug recommendations. GAT-ETM code is available at https://github.com/li-lab-mcgill/GAT-ETM.

## Introduction

The rapid growth in volume and diversity of electronic health records (EHR) has enabled health informatics research to refine disease phenotypes and discover novel disease comorbidities. Modern hospitals routinely generate standardized EHR observations such as the International Classification of Diseases (ICD) for diagnoses, Drug Identification Number (DIN) for prescription, and Anatomical Therapeutic Chemical (ATC) for drug ingredients. Specifically, ICD is a widely used health care classification system to classify diseases, symptoms, signs, abnormal findings, social circumstances, complaints and external causes of injury or disease. A DIN (Canada) or NDC (USA) code uniquely identifies all approved pharmaceutical products sold in dosage forms in Canada. Anatomical Therapeutic Chemical (ATC) is a medicine classification system maintained by the World Health Organization (WHO). Each ATC code is specific to an active drug ingredient, which is indicative of the patient’s health state. The rich patient EHR information enables computational phenotyping^1^, risk prediction^2^, patient stratification^3^, and patient similarity analysis^4^.

Extracting meaningful medical concepts by modeling the joint distribution of the EHR data is challenging due to its large feature space. Among diverse machine learning approaches, topic models provide an efficient way to exploit sparse and discrete data. They were originally developed to identify word occurrence patterns in text corpus^5^. A topic model infers a set of categorical distributions over the vocabulary, called latent topics, and represents each document by a topic mixture. In their applications to EHRs, we treat each patient’s clinical history as a document and each EHR observation (e.g. ICD code) as a word within its document. Our goal then is to learn clinically meaningful phenotype topics and disease mixture memberships of patients. Recently, several topic models were developed to effectively infer latent topics from EHR data^6–9^. However, these methods usually perform poorly in the modeling of rare administrative codes due to insufficient observations of them, which results in under-representation of these codes among the inferred topic distributions.

In this paper, we present a neural topic model called Graph-ATtention Embedded Topic Model (GAT-ETM). To capture higher-level medical concepts, GAT-ETM uses a graph attention network (GAT)^10^ to compute the embeddings of EHR codes from a taxonomy graph of the relations between and within the disease-code and drug-code modalities via the multi-head attention mechanism. The resulting EHR code embeddings are then used to infer a set of coherent multimodal topics from the patient-level EHR data via the Embedded Topic Model (ETM)^11^. Learning of the embeddings of the EHR codes and the latent topics are performed simultaneously in an end-to-end fashion without supervision. We evaluated GAT-ETM on a large-scale EHR dataset consisting of administrative records for 1.2 million patients from Quebec, Canada. GAT-ETM demonstrated high-quality topic inference and accurate drug imputation.

## Related methods and our contributions

Recently, many automatic EHR-based phenotyping algorithms were developed using rule-based^12–14^ or machine learning techniques^15–21^. MixEHR^6^ extended latent Dirichlet Allocation (LDA)^5^ to multimodal topic inference to account for the heterogeneous nature of EHR data distributions. However, MixEHR is unable to make use of knowledge graphs. In order to achieve better performance in modelling noisy and sparse EHR data, several recent methods are able to utilize medical knowledge graphs. For instance, GRAM^22^ and KAME^23^ employed attention mechanism to incorporate medical knowledge into clinical modelling. GRAM considers taxonomic hierarchy as a knowledge prior and generates representation of medical concepts for a predictive task. KAME only used the medical knowledge related to the last visit in a recurrent neural network (RNN). RETAIN^24,25^ is a two-level attention model that detects influential past visits and crucial clinical variables within those visits. DG-RNN^26^ employed an attention module and uses long short-term memory (LSTM) to models sequential medical events. To handle various healthcare tasks, TAdaNet^27^ a meta-learning model makes use of a domain-knowledge graph to provide task-specific customization. These recent models are mostly focused on supervised learning tasks, and therefore their learning algorithms require labelled data. A recent model called Graph Embedded Topic Model (GETM)^28^ leveraged a knowledge graph by combining node2vec^29^ with embedded topic model (ETM)^30^ in a pipeline approach. GETM is an unsupervised model that directly learns the distribution of the EHR data using the node2vec embedding. However, because the graph embedding is learned separately from the EHR modeling task, it may not always help in learning the EHR data.

**Table 1:** Notation definitions.

Notations	Descriptions
*D*	# of patients in the dataset
*K*	# of topics
$c_{pn}^{(t)}$	The *n*th code of type *t* of patient *p*
$V_{\text {icd}}, V_{\text {atc}}$	Size of ICD, ATC vocabulary, respectively
$N_{pt}$	# of observed EHR codes of type *t* for patient *p*
${\mathbf {v}}_p\in \mathbb {N}^{V_{\text {icd}}+V_{\text {atc}}}$	Observed code frequency for patient *p*
${\varvec{\uptheta }}_p\in S^{K-1},$	Topic mixture of patient *p*, $\sum _k{\varvec{\uptheta }}_{pk}=1$
$\varvec{\uprho }^{\text {(icd)}}\in \mathbb {R}^{L \times V_{\text {icd}}}$	KG-informed embedding of ICD codes
$\varvec{\uprho }^{\text {(atc)}} \in \mathbb {R}^{L \times V_{\text {atc}}}$	KG-informed embedding of ATC codes
$\varvec{\upalpha }^{\text {(icd)}} \in \mathbb {R}^{L\times K}$	Embedding of topics for ICD code
$\varvec{\upalpha }^{\text {(atc)}} \in \mathbb {R}^{L\times K}$	Embedding of topics for ATC code
$\varvec{\upbeta }_k^{\text {(icd)}} \in S^{V_{\text {icd}}-1}$	*k*th ICD topic distribution, $\sum ^{V_{\text {icd}}}_{v=1}\varvec{\upbeta }_{kv}^{\text {(icd)}} = 1$
$\varvec{\upbeta }_k^{\text {(atc)}} \in S^{V_{\text {atc}}-1}$	*k*th ATC topic distribution, $\sum ^{V_{\text {atc}}}_{v=1}\varvec{\upbeta }_{kv}^{\text {(atc)}} = 1$

In contrast to the existing works, our contributions are 3-fold:

1. GAT-ETM is an end-to-end neural topic framework, which simultaneously learns the medical code embedding from a medical knowledge graph of diseases (ICD-9 code) and drugs (ATC code) and the topic embedding from EHR data;
2. To extract meaningful and interpretable disease topics, we use a linear decoder to reconstruct EHR data such that the linear projections can directly map to individual latent topics; and,
3. To maximize information flow among the EHR nodes on the graph, we proposed a graph augmentation strategy by connecting nodes with their ancestry nodes along the taxonomy; we combine the two knowledge graphs (of ICD-9 and ATC) via known disease-drug links (i.e. drug treatments for diseases), which allows information sharing between the two data types during the training.

## Methods

### Notations

We denote the number of patients, the number of topics, the size of ICD vocabulary, and the size of ATC vocabulary as *D*, *K*, $V_{\text {icd}}$, $V_{\text {atc}}$, respectively. For a patient *p*, $c_{pn}^{(t)}$ denotes the *n*th code of type $t\in \{{\text {ICD}}, {\text {ATC}}\}$. Another way to express the EHR history of patient *p* is via a $(V_{\text {icd}}+V_{\text {atc}})$-dimensional frequency vector ${\mathbf {v}}_p$. ${\varvec{\uptheta }}_p$ denotes a *K*-dimensional probabilistic mixture membership over *K* disease topics that sum to 1. For the *k*th topic, $\varvec{\upbeta }^{(t)}_k$ denotes the code distribution for the ICD or ATC modality. The topic embedding weights are denoted by $L\times K$ matrices ${\varvec{\varvec{\upalpha }}}^{\text {(icd)}}$ and ${\varvec{\varvec{\upalpha }}}^{\text {(atc)}}$, where *L* is the dimension of latent embedding space. The knowledge graph (KG)-informed embedding of medical codes of type $t\in \{{\text {icd}}, {\text {atc}}\}$ is denoted by a $L\times V_{t}$ matrix ${\varvec{\varvec{\uprho }}}^{(t)}$. Note that the L-dimensional embedding is shared among topics, ICD codes, and ATC codes. Table 1 lists the key notations.

### Generative process

**Figure 1:**
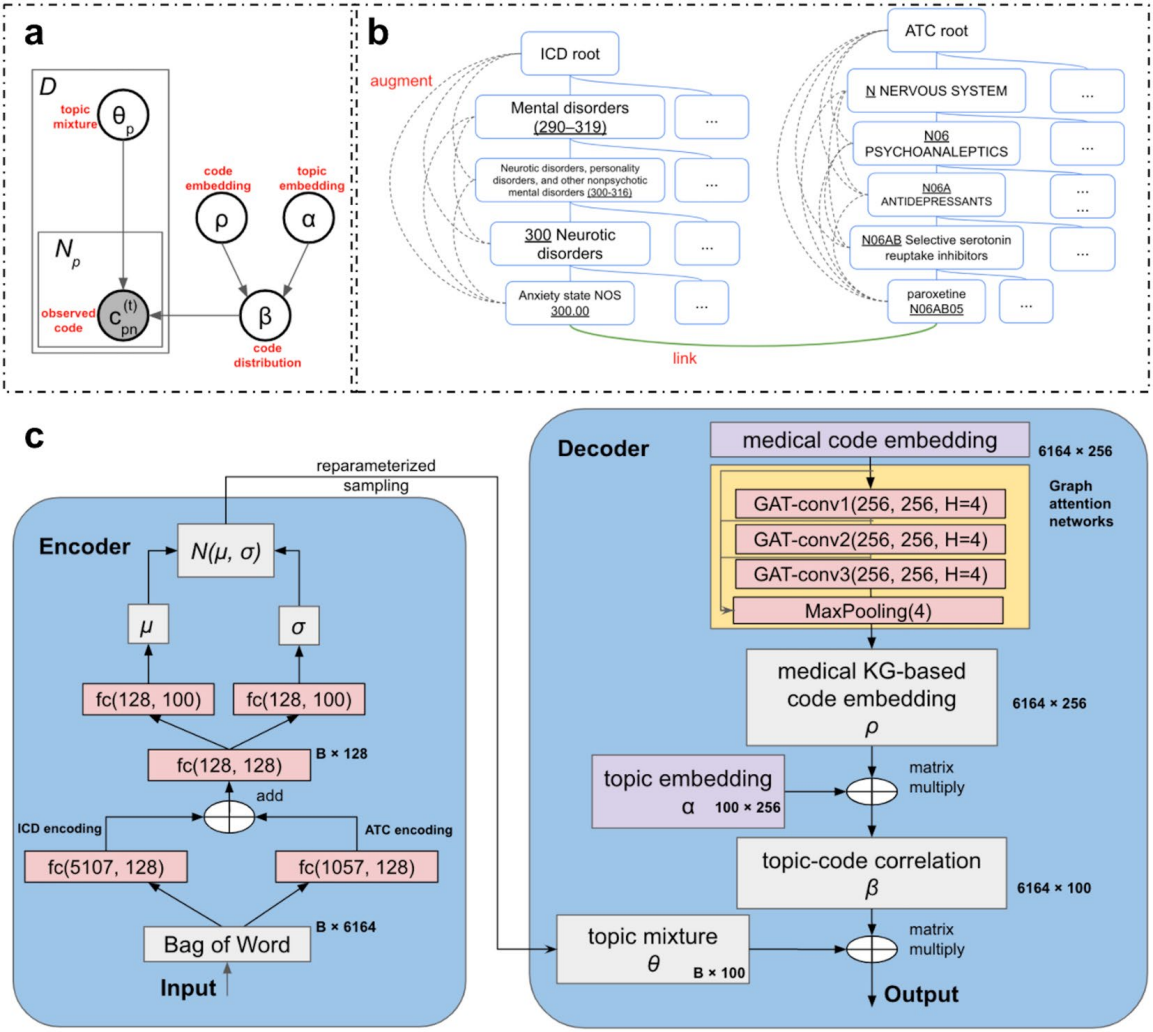
GAT-ETM model overview. (**a**) The probabilistic graphical model view of GAT-ETM. (**b**) The augmentation and merger applied on the taxonomy knowledge graphs. (**c**) The illustration of the deep learning architecture used to perform variational inference over the GAT-ETM model.

GAT-ETM assumes the following generative process (Fig. 1a):

For each patient $p \in \{1,\ldots ,D\}$:

1. Draw topic mixture membership ${\varvec{\uptheta }}_p \sim \mathcal{LN}\mathcal{}(0, I)$:
  $$\begin{aligned} {\varvec{\varvec{\updelta }}}_p&\sim {\mathcal {N}}(\mathbf {0},\mathbf {I}),\quad \mathbf {\varvec{\uptheta }}_p = \frac{\exp (\varvec{\varvec{\updelta }}_p)}{\sum _{k'}\exp (\varvec{\updelta }_{pk'})} \end{aligned}$$
2. For each EHR code $c_{pn}^{(t)}$, $t\in \{{\text {icd}},{\text {atc}}\}$:
  $$\begin{aligned} c^{(t)}_{pn}\sim Cat(\varvec{\varvec{\upbeta }}^{(t)}\varvec{\uptheta }_p). \end{aligned}$$

where $\mathcal{LN}\mathcal{}$ and *Cat* stand for logistic-normal and categorical distribution, respectively. The *k*th topic distribution $\varvec{\varvec{\upbeta }}^{(t)}_k$ is defined by the inner product of the code embedding $\varvec{\varvec{\uprho }}^{(t)}$ and topic embedding of the *k*th topic $\varvec{\varvec{\upalpha }}_{\cdot k}$:

1 $$\begin{aligned} \varvec{\varvec{\upbeta }}^{(t)}_k = \text {softmax}({\varvec{\varvec{\uprho }}^{(t)}}^\intercal \varvec{\varvec{\upalpha }}_{\cdot k}) = \frac{\exp ({\varvec{\varvec{\uprho }}^{(t)}}^\intercal \varvec{\varvec{\upalpha }}_{\cdot k})}{\sum _v\exp ({\varvec{\varvec{\uprho }}_{v.}^{(t)}}^\intercal \varvec{\varvec{\upalpha }}_{\cdot k})} \end{aligned}$$

where ${\varvec{\varvec{\uprho }}^{(t)}_{v.}}^\intercal$ is the $1 \times L$ row embedding of code *v* of type *t* and $\varvec{\varvec{\upalpha }}_{\cdot k}$ is the $L\times 1$ column embedding of topic *k*. Inner product, as a similarity metric, indicates the relevance between codes and the topic.

### Evidence lower bound

The marginal log-likelihood of the EHR corpus is:

2 $$\begin{aligned} \log p(\mathbf {V} \mid \varvec{\uprho },\varvec{\upalpha })&= \sum _{p=1}^D \int \log p(\varvec{\uptheta }_p) p({\mathbf {v}}_p \mid \varvec{\uptheta }_p, \varvec{\uprho },\varvec{\upalpha }) \text {d}\varvec{\uptheta }_p \nonumber \\&= \sum _{p=1}^D \int \log p(\varvec{\uptheta }_p) \text {d}\varvec{\uptheta }_p + \sum _{t\in \{{\text {icd}}, {\text {atc}}\}}\sum ^{N_{pt}}_{n=1} \log \varvec{\upbeta }_{c_{pn}^{(t)}\cdot }^{(t)} \varvec{\uptheta }_p \text {d}\varvec{\uptheta }_p \end{aligned}$$

which involves an intractable integral over the K-dimensional latent topic mixture $\varvec{\uptheta }_p$ for every patient. To approximate the log-likelihood, we took an variational autoencoder (VAE) approach using a variational Gaussian $q(\varvec{\uptheta }_p \mid {\mathbf {v}}_p, {\mathbf {W}})$, which is parameterized by a set of neural network parameters ${\mathbf {W}}$^31^. We optimize the network parameters ${\mathbf {W}}$ by maximizing the following evidence lower bound (ELBO):

3 $$\begin{aligned} \log p(\mathbf {V} \mid \varvec{\uprho },\varvec{\upalpha })&\ge \sum _p \mathbb {E}_{q(\varvec{\uptheta }_p; {\mathbf {v}}_p, {\mathbf {W}})}\left[ \log p({\mathbf {v}}_p | \varvec{\uptheta }_p, \varvec{\uprho }, \varvec{\upalpha })\right] \nonumber - \sum _p {\text {KL}}\left[ q(\varvec{\uptheta }_p \mid {\mathbf {v}}_p, {\mathbf {W}}) || p(\varvec{\uptheta }_p)\right] \nonumber \\&\equiv ELBO({\mathbf {W}},\varvec{\uprho },\varvec{\upalpha }) \end{aligned}$$

where the first term is the approximated log-likelihood and the second term is the KL divergence between the proposed variational and the prior for $\varvec{\uptheta }_p$.

### Inferring patients’ topic mixture

To infer $q(\varvec{\uptheta }_p \mid {\mathbf {v}}_p, {\mathbf {W}})$ using VAE, we have the following encoder architecture. Given an EHR document of two data types ${\mathbf {v}}_p=[{\mathbf {v}}_p^{\text {(icd)}} || {\mathbf {v}}_p^{\text {(atc)}}]$, the encoder has two input layers with rectified linear unit (ReLU) activation functions that separately encode ${\mathbf {v}}_p^{\text {(icd)}}$ and ${\mathbf {v}}_p^{\text {(atc)}}$ with two 128-dimensional vectors $\mathbf {e}_p^{\text {(icd)}}$ and $\mathbf {e}_p^{\text {(atc)}}$. We then perform element-wise addition of the encoding vectors. The resulting 128-dimensional vectors is passed to a two fully-connected feedforward functions ${\textbf {NN}}_{\varvec{\upmu }}$ and ${\textbf {NN}}_{\varvec{\upsigma }}$ to generate the mean and $\log$ standard deviation of the proposed distribution $q(\varvec{\uptheta }_p \mid {\mathbf {v}}_p, {\mathbf {W}})$ for patient *p*:

4 $$\begin{aligned} \varvec{\upmu }_p&= {\textbf {NN}}_{\varvec{\upmu }}({\textbf {e}}_p^{\text {(icd)}}+{\textbf {e}}_p^{\text {(atc)}}; {\mathbf {W}}_{\varvec{\upmu }}), \end{aligned}$$

5 $$\begin{aligned} \log \varvec{\upsigma }_p&= {\textbf {NN}}_{\varvec{\upsigma }}({\textbf {e}}_p^{\text {(icd)}}+{\textbf {e}}_p^{\text {(atc)}}; {\mathbf {W}}_{\varvec{\upsigma }}) \end{aligned}$$

### Learning medical code embedding from knowledge graph

We leverage an ICD-ATC knowledge graph to learn code embedding $\varvec{\uprho }^{\text {(icd)}}, \varvec{\uprho }^{\text {(atc)}}$. As shown in Fig. 1b, there are 3 types of relations in this knowledge graph: (1) ICD hierarchy (https://icdlist.com/icd-9/index) augmented by linking each pair of ancestral nodes and child nodes, (2) ATC hierarchy (https://www.whocc.no/atc_ddd_index/) augmented by linking each pair of descendants and ancestors, and (3) ICD-ATC relations (http://hulab.rxnfinder.org/mia/). We extracted these relations from their corresponding websites and constructed an undirected knowledge graph ${\mathcal {G}}=\{{\mathcal {V}}, {\mathcal {E}}\}$, where ${\mathcal {V}}$ contains all of the ICD and ATC codes as the nodes and ${\mathcal {E}}$ contains ICD-ICD, ATC-ATC, and ICD-ATC relations as the edges.

The resulting knowledge graph is sparsely connected because of the tree-structure of both ICD and ATC taxonomy. To further improve the information flow, we augmented the knowledge graph by connecting each node to all of its ancestral nodes (Fig. 1b).

To learn the node embedding, we used a GAT^10^ (Fig. 1c). We chose GAT among other graph neural networks (GNNs) because of its flexibility to represent each node by its neighbor via the multi-head self-attention mechanism. Specifically, we first initialize the embedding $\varvec{\uprho }^{(0)}$ by training a node2vec model^29^ on the knowledge graph with embedding dimensions set to 256. We then feed the resulting embedding as the initial embedding to a multi-layer GAT, which computes the embedding at the *i*th layer as:

6 $$\begin{aligned} \varvec{\uprho }_c^{(i)}&= \sum _{c' \in \{c\}\cup {\mathcal {N}}(c)} w_{cc'}^{(i)} {\mathbf {W}}_i \varvec{\uprho }_{c'}^{(i-1)} \end{aligned}$$

where ${\mathcal {N}}(c)$ denotes the neighbor nodes of node *c* and the attention coefficients $w_{cc'}^{(i)}$ is computed as:

7 $$\begin{aligned} w_{cc'}^{(i)}= \frac{\exp ({\text {LeakyReLU}}({\mathbf {a}_i}^T[ {\mathbf {W}}_i\varvec{\uprho }_c^{(i)}|| {\mathbf {W}}_i\varvec{\uprho }_{c'}^{(i)}])}{\sum _{j \in \{c\}\cup {\mathcal {N}}(c)}\exp ({\text {LeakyReLU}}({\mathbf {a}_i}^T[ {\mathbf {W}}_i\varvec{\uprho }_c^{(i)}|| {\mathbf {W}}_i\varvec{\uprho }_{j}^{(i)}])} \end{aligned}$$

where $\mathbf {a}_i, {\mathbf {W}}_i$ are the parameters of the *i*th layer of the GAT network. The output of all the layers are max-pooled to produce a $L \times V$ embedding matrix denoted as $\varvec{\uprho }=[\varvec{\uprho }^{\text {(icd)}}||\varvec{\uprho }^{\text {(atc)}}]$, which is used as the EHR code embeddings in Eq. (1).

### Learning procedure

In the above model, we have a set of learnable parameters including the VAE encoder network parameters ${\mathbf {W}}_{\theta }$ for $q(\varvec{\uptheta }_p \mid {\mathbf {v}}_p, {\mathbf {W}}_{\theta })$, the GAT network parameters ${\mathbf {W}}_{\rho }$ for generating the code embedding $\varvec{\uprho }$ , and the fixed point topic embedding $\varvec{\upalpha }$. To learn them, we maximize the ELBO (Eq. 3) with respect to those parameters. Specifically, we used stochastic optimization, forming noisy gradients by taking Monte Carlo approximations of the expected gradient through the re-parameterization trick^31^:

8 $$\begin{aligned}{}&ELBO({\mathbf {W}}_{\theta }, {\mathbf {W}}_{\rho }, \varvec{\uprho }, \varvec{\upalpha }) \approx \sum _{p\in {\mathcal {B}}} \left[ \log p({\mathbf {v}}_p | \hat{\varvec{\uptheta }}_p, \varvec{\uprho }, \varvec{\upalpha })\right] - \sum _{p\in {\mathcal {B}}} \log q(\hat{\varvec{\updelta }}_p \mid {\mathbf {v}}_p, {\mathbf {W}}_{\theta }) + \log p(\hat{\varvec{\updelta }}_p) \end{aligned}$$

where $$\hat{\varvec{\updelta }}_p \sim \varvec{\upmu }_p + \varvec{\upsigma }_p{\mathcal {N}}(0,I)$$ and $$\hat{\varvec{\uptheta }}_p = {\text {softmax}}(\hat{\varvec{\updelta }}_p)$$.

To handle large EHR data collection, we use mini-batch stochastic gradient decent to update the model with each mini-batch of size $|{\mathcal {B}}|<< D$^32^. Algorithm 1 summarizes the GAT-ETM learning procedure.
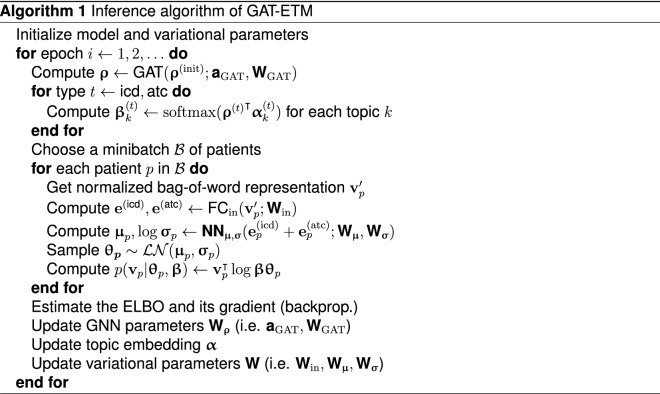


### Implementation details

We used the Adam optimizer to train GAT-ETM. The learning rate was set as 0.01. We use L2 regularization on the variational parameters. The weight decay parameter is $1.2 \times 10^{-6}$. The minibatch size is 512. The embedding size of the topic and code embedding was set to 256. The embedding size in the inference encoder was set to 128. Empirically we found that the number of GAT layers being 3 and number of heads being 4 gave good performance although GAT-ETM was fairly robust to these hyperparameter settings. We trained the model until convergence as determined by the marginal improvement of the ELBO.

### Data processing

To evaluate our model, we used a real-world large EHR database called Population Health Record (PopHR), which was originally created for monitoring population health from multiple distributed sources^33,34^. PopHR contains longitudinal administrative data of 1.2 million patients with up to 20-years of follow-up. For each patient, we collapsed the time series data to obtain the frequency of distinct EHR codes observed over his or her entire medical history (i.e., ${\mathbf {v}}_p$). We treated the frequency as an EHR document. We started with two types of EHR data: (1) 5107 unique ICD-9 codes, and (2) over 10,000 DIN codes. Since different DIN codes may indicate the same ingredient(s) of different strength(s), we converted the DIN codes to 1057 ATC codes according to their ingredient(s).

For disease classification tasks, we obtained gold-standard labels for 9 chronic diseases using the corresponding rules defined by the Chronic Disease Surveillance Division of the Public Health Agency of Canada^35^. These include acute myocardial infarction (AMI), asthma, congestive heart failure (CHF), COPD, diabetes, hypertension, ischemic heart disease (IHD), epilepsy, and schizophrenia. Additionally, we constructed gold-standard labels for ADHD, HIV, and Autism based on the disease definitions described in^36,37^, and Autism Spectrum Disorder Surveillance in Quebec report^38^, respectively. Therefore, we obtained in total 12 phenotypes where we have rule-based labels to evaluate the classification accuracy of a given model as detailed in “3.9 Phenotype classification task” section.

### Evaluation metrics

#### Reconstruction

We conducted a document completion task and calculated log-likelihood as the metric of predictive capacity. We split the PopHR dataset into 60% training, 30% validation, and 10% test. We randomly divided each test EHR document into halves. We used the first half to predict the expected topic mixture of the test patient ($\bar{\varvec{\uptheta }}_p = {\text {softmax}}(\varvec{\upmu }_p$)) and the other half to evaluate the predicted log-likelihood on the held-out EHR tokens.

#### Topic quality

Since the interpretation of the topics learned by the model is also crucial, For every inferred topic distribution, we computed their topic quality score^11^, which is the product of topic coherence and topic diversity. Topic coherence^39^ measures the observed co-occurrence rate of the top codes within the same topic for every topic. It is defined as the average pointwise mutual information of two codes drawn randomly from the same document:

9 $$\begin{aligned} {\mathrm {TC}}=\frac{1}{K} \sum _{k=1}^{K} \frac{2}{s(s-1)} \sum _{1\le i\le j\le s} \frac{\log \frac{P(w_i^{(k)}, w_j^{(k)})}{P(w_i^{(k))} P(w_j^{(k)}}}{-\log P(w_i^{(k)},w_j^{(k)})}, \end{aligned}$$

where $\{w_1^{(k)},\ldots ,w_s^{(k)}\}$ denotes the top-*s* codes with the highest probability in topic *k*, $P(w_i^{(k)}, w_j^{(k)} )$ is the probability of words $w_i^{(k)}$ and $w_j^{(k)}$ co-occurring in an EHR document and $P(w_i^{(k)})$ is the marginal probability of code $w_i^{(k)}$. Topic diversity^11^ measures the uniqueness of across topics, which reflects the model’s ability to capture the phenotypic diversity. It is defined as the percentage of unique codes in the top-*r* codes across all topics:

10 $$\begin{aligned} {\mathrm {TD}}&= \frac{1}{Kr} {\mathrm {unique}}\left( \bigcup _{k=1}^{K} \bigcup _{i=1}^r \left\{ w_i^{(k)}\right\} \right) . \end{aligned}$$

where ${\mathrm {unique}}(\cdot )$ is the function to count the number of unique elements in a set. Topic quality (TQ) is defined as TC $\times$ TD. In our evaluation, we set $s=3, r=3$ for the calculation of TC and TD, respectively. We measured TQ for ICD codes and ATC codes separately and then computed their average.

#### Phenotype classification task

We used the phenotype labels generated from the rules as gold-standards to evaluate our models (“Data processing ” section). We split the dataset into 72%, 8%, and 20% for training, validation, and test, respectively. We first trained an unsupervised model to infer the patients’ topic mixture membership from the training set. We then trained a LASSO classifier using patients’ topic mixture $\varvec{\uptheta }_p$ as input features to predict phenotype labels for each phenotype using the training set. We chose the lambda penalty in LASSO from a range between 0.01 and 1 using the validation set. For the test data, we first used the trained unsupervised model to infer the test patients’ topic mixture and then used the trained LASSO to predict their phenotype labels. We evaluated the models by Area Under the Receiver Operating Characteristic curve (AUROC). Higher AUROC implies more informative phenotype topic mixture derived by the corresponding unsupervised method. We repeated the experiments 10 times to obtain standard deviation of the AUROC estimates for each method, each time with a different random split of data into the training, validation, and testing set.

#### Drug imputation task

We sought to impute ATC codes based only on ICD codes. We focused on drug imputation because it has more practical applications as a drug recommender system, i.e., predicting drugs based on patient diagnoses. Specifically, we first inferred $\hat{\varvec{\uptheta }}_p$ from input EHR of patient *p* using only the ICD codes. We then inferred the expectation of each ACT code $\hat{c}_{pv}^{\text {(atc)}} = \varvec{\upbeta }_v^{(\text {atc})}\hat{\varvec{\uptheta }}_p$.

We evaluated the models by patient-wise accuracy and drug-wise accuracy. For patient-wise accuracy, we compared the precision, recall, and F1-score of the top-5 predictions averaged over all patients (prec@5, recall@5, F1-score@5). In both training and test datasets, patients with less than 5 ATC codes were filtered out.

The drug-wise accuracy measures the imputation accuracy at of different observed frequency. Specifically, we sorted and binned the ATC codes into five frequency quantiles, where 0–20% contains the rarest ATC codes and 80–100% contains the most frequently observed ATC codes. We then computed the recall on each ATC code and took the average (weighted by frequency) of the codes in each bin. We then computed the top-30 precision (i.e., true positive divided by predicted positive) at each quantile for each method.

### Baselines

We compared the performance of GAT-ETM  with the following baseline approaches:

- MixEHR^6^ is a generative multimodal topic model. We considered it as a baseline because it was developed to deal with EHR data of high sparsity, bias, and heterogeneity but uses strong mean-field assumption to perform varaitional inference of the latent topic distributions.
- ETM^30^ is a topic model that introduces feature embedding of words and topics. We considered it as a baseline because it has a similar generative process as GAT-ETM but does not utilize knowledge graph.
- GETM^28^ is an embedded topic model that leverages ICD and ATC medical taxonomy hierarchies by initializing word embedding as the output of node2vec. Note that GETM obtains code embedding on only ICD and ATC taxonomy hierarchies respectively. It neither connects ICD and ATC taxonomy together nor does it conduct augmentation. We considered GETM as the baseline because it harnesses external medical knowledge graphs although not in an end-to-end manner.

Based on empirical study, we set the number of topics *K* as 100 for the baseline models and our model and the number of embedding dimensions as 256 for embedding-based methods (i.e. ETM, GETM, and GAT-ETM). The number of layers of inference networks (i.e., the encoder) was set to be 3 for ETM and GETM to fairly compared with ours.

For drug imputation, we also evaluated two traditional approaches:

- Frequency-based model: we counted the occurrence of all ATC codes in the training data, and then imputed the most frequent codes for the test patients.
- K nearest neighbors: for each patient in the test set, we found K nearest neighbors according to its frequency vector ${\mathbf {v}}_p$. We then averaged the ATC codes of the nearest neighbors as the ATC predictions for the test patients. We selected the optimal number of neighbors $K \in \{100, 200, 500, 1000, 5000\}$ and the best distance metrics $\in \{manhattan, minkowski\}$ using the validation set.

### Ablation study

An ablation study was conducted to evaluate the three key features of GAT-ETM:

1. *Initialization of code embedding*: when this procedure is discarded, we randomly initialized the embedding for GAT rather than pre-trained them by node2vec.
2. *Augmentation of knowledge graph*: when this procedure is discarded, we did not connect each node with all of its ancestors.
3. *Graph attention networks*: when this module is discarded, we fixed the code embedding that is generated by node2vec. In other word, it is equivalent to GETM with the augmented, merged knowledge graph.

## Results

### Reconstruction and topic quality

As shown in Table 2, GAT-ETM performed the best on both likelihood and topic quality. In terms of reconstruction and topic quality, MixEHR performed similarly to ETM but notably worse compared with GETM and GAT-ETM. The superior performance of the neural topic models over the statistical framework in MixEHR may be attributable to the flexibility of the deep learning frameworks in capturing the EHR code embedding. Also as we expected, ETM performed worse than knowledge graph-based models under every metric possibly because of its inadequacy in modeling sparse and noisy EHR data without leveraging the graph information. Compared to GAT-ETM, GETM achieved higher TD but lower TC, which means that the topic distributions over the EHR codes are more diverse but less coherent with the PopHR dataset. Indeed, GETM learns the code embedding only from the separate knowledge graphs and then fixes it during the ETM training on the EHR dataset. In contrast, GAT-ETM utilized a GAT to flexibly fine-tune the node2vec-pretrained code embedding simultaneously while modeling the EHR dataset. This led to higher TC and higher overall TQ and better reconstruction performance compared to GETM.

Table 3 summarizes the results of the ablation study. All of the three novel features we introduced to the original GETM conferred notable improvements on the prediction performance and topic quality. Considering log-likelihood, the graph augmentation improved the predictive power of our model the most, the GAT module that enables the end-to-end training manner came the second. Considering TQ, pre-training code embedding took the most crucial role. Compared with GETM’s performance in terms of reconstruction loss (184.32) and TQ (0.1843) in Table 2, we found that GETM with graph augmentation achieved lower reconstruction loss (180.44) but worse TQ (0.1768). It is possibly due to the fact that the connection between medical concepts are not the same but with different type and weight. This founding highlights the importance of using the GAT to assign different attention to edges.

**Table 2:** Reconstruction loss and topic quality. (Recon., Reconstruction error on the held-out EH data; NLL., negative log-likelihood on the held-out data. Both are the lower the better.).

Model	Recon.	Topic quality [ICD,ATC]			
	NLL.	Topic coherence	Topic diversity	Topic quality	TQ (ave.)
MixEHR^6^	203.97	0.109, 0.264	0.307, 0.383	0.0335, 0.1011	0.0673
ETM^11^	198.26	0.113, 0.233	0.373, 0.423	0.0421, 0.0986	0.0704
GETM^28^	184.32	0.167, 0.271	**0.86**, **0.83**	**0.1436**, 0.2249	0.1843
GAT-ETM (proposed)	**172.69**	**0.18**, **0.314**	0.76, 0.787	0.1368, **0.2471**	**0.1920**

The best score for each metric are in [bold].

**Table 3:** Ablation study.

Model	Recon.	Topic quality [ICD,ATC]			
	NLL.	Topic coherence	Topic diversity	Topic quality	TQ (ave.)
GAT-ETM	**172.69**	**0.18**, **0.314**	0.76, 0.787	**0.1368**, **0.2471**	**0.1920**
GAT-ETM (w/o init.)	179.59	0.139, 0.193	0.573, 0.447	0.0796, 0.0863	0.0830
GAT-ETM (w/o aug.)	181.63	0.162, 0.282	0.733, 0.75	0.1187, 0.2115	0.1651
GETM (w/ aug.)	180.44	0.161, 0.282	**0.783**, **0.807**	0.1261, 0.2276	0.1768

The best score for each metric are in [bold].

### Phenotype classification task

**Figure 2:**
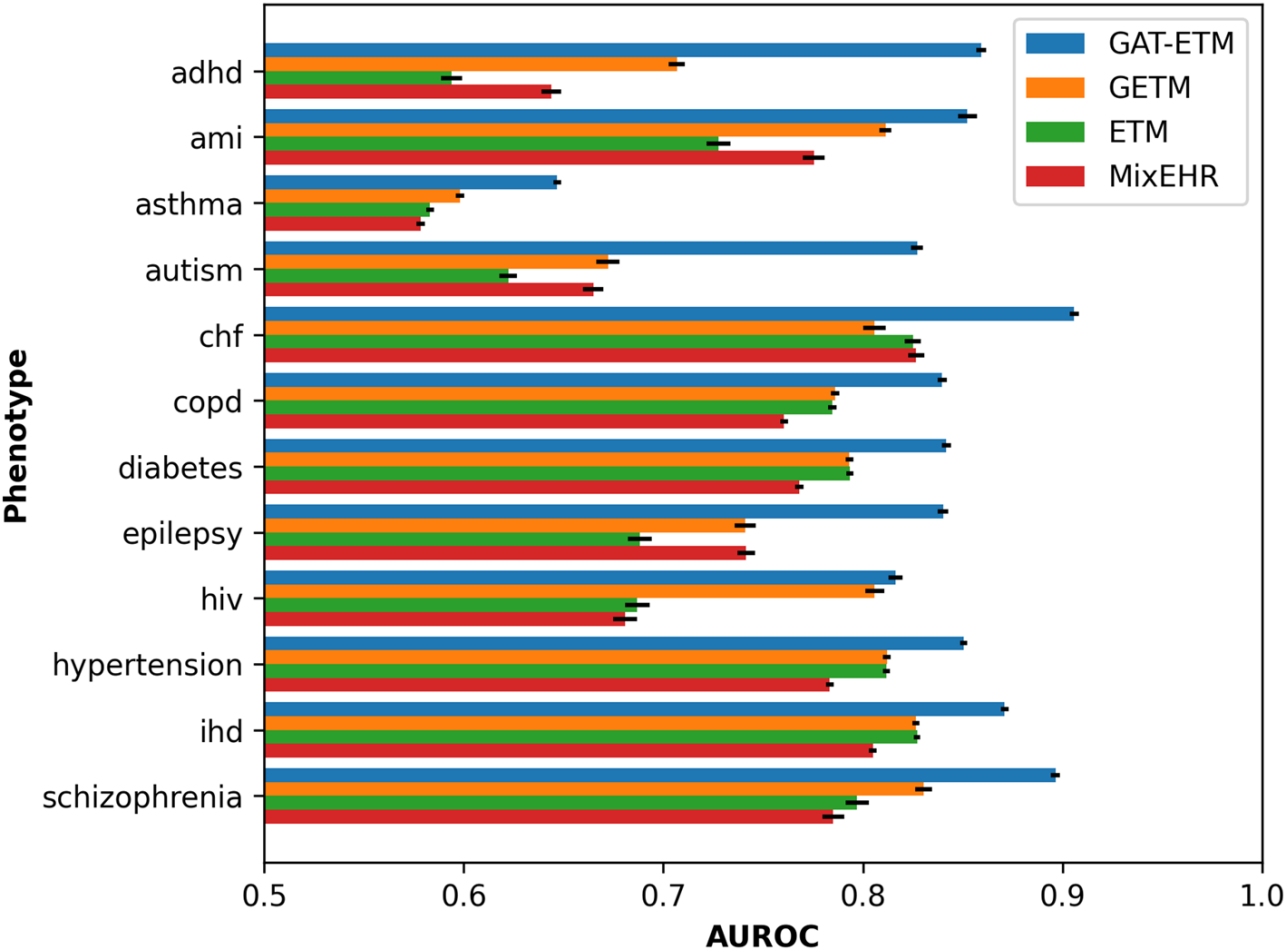
The classification performance of the phenotypes using expert-derived rule-based labels. We applied GAT-ETM and three baseline unsupervised phenotyping methods namely, GETM, ETM, and MixEHR, to the PopHR data without supervision. For each phenotype, we trained a LASSO classifier using patients’ topic mixture as features. The barplots display average Area Under the Receiver Operating Characteristic Curve (AUROC) treating the rule-based phenotype labels as gold-standards. The error bar indicates standard deviation of 10 repeated experiments, each time with different random split of the training, validation, and testing data.

We further evaluated each method by phenotype classification on 12 rule-based phenotypes (“Phenotype classification task” section). GAT-ETM achieved the most accurate classification performance in terms of AUROC on all 12 chronic diseases (Fig. 2). GETM conferred higher or comparable performance compared with ETM, which reflects the value of leveraging knowledge graph information. Therefore, GAT-ETM is able to generate informative patient latent embeddings for the 12 automatic phenotyping tasks.

**Table 4:** Patient-wise imputation measurement.

Model	Prec@5	Recall@5	F1-score@5
Frequency-based model	0.1049	0.0432	0.0577
K nearest neighbor	0.1606	0.0713	0.0930
ETM	0.1823	0.0833	0.1075
GETM	0.2378	0.1101	0.1418
GAT-ETM	** 0.2600**	**0.1225**	**0.1569**

The best score for each metric are in [bold].

**Table 5:** Top-30 drug-wise imputation precision at different percentiles of drug frequencies.

Model	Percentile of frequencies				
	20–40	40–60	60–80	80–100	Ave.
ETM	0.0039	0.0188	0.0479	0.3847	0.3058
GETM	0.0213	0.0542	0.0934	0.4352	0.3597
GAT-ETM	**0.0345**	**0.0841**	**0.1239**	**0.4583**	**0.3815**

The best score for each metric are in [bold].

### Drug imputation task

We next evaluated the model performance in terms of drug imputation accuracy (“Drug imputation task” section). Table 4 shows the result of patient-wise imputation performance. GAT-ETM achieved the highest scores on all 3 metrics. In terms of drug-wise imputation precision, GAT-ETM also outperformed both baselines (Table 5). Specifically, compared to ETM, GAT-ETM’s precision@30 is 9 times better at 20–40%, 5 times better at 40–60%, 3 times better at 60–80%, and 25% higher at 80–100% quantiles of observed frequencies. Compared to GETM, GAT-ETM’s precision@30 is 62% better on 20–40%, 55% better on 40–60%, 33% better on 60–80%, 5% better on 80–100%. This indicates that by flexibly leveraging the knowledge graph via embedding learning, GAT-ETM conferred higher precision to drug imputation especially in predicting low frequently observed ATC codes.

**Figure 3:**
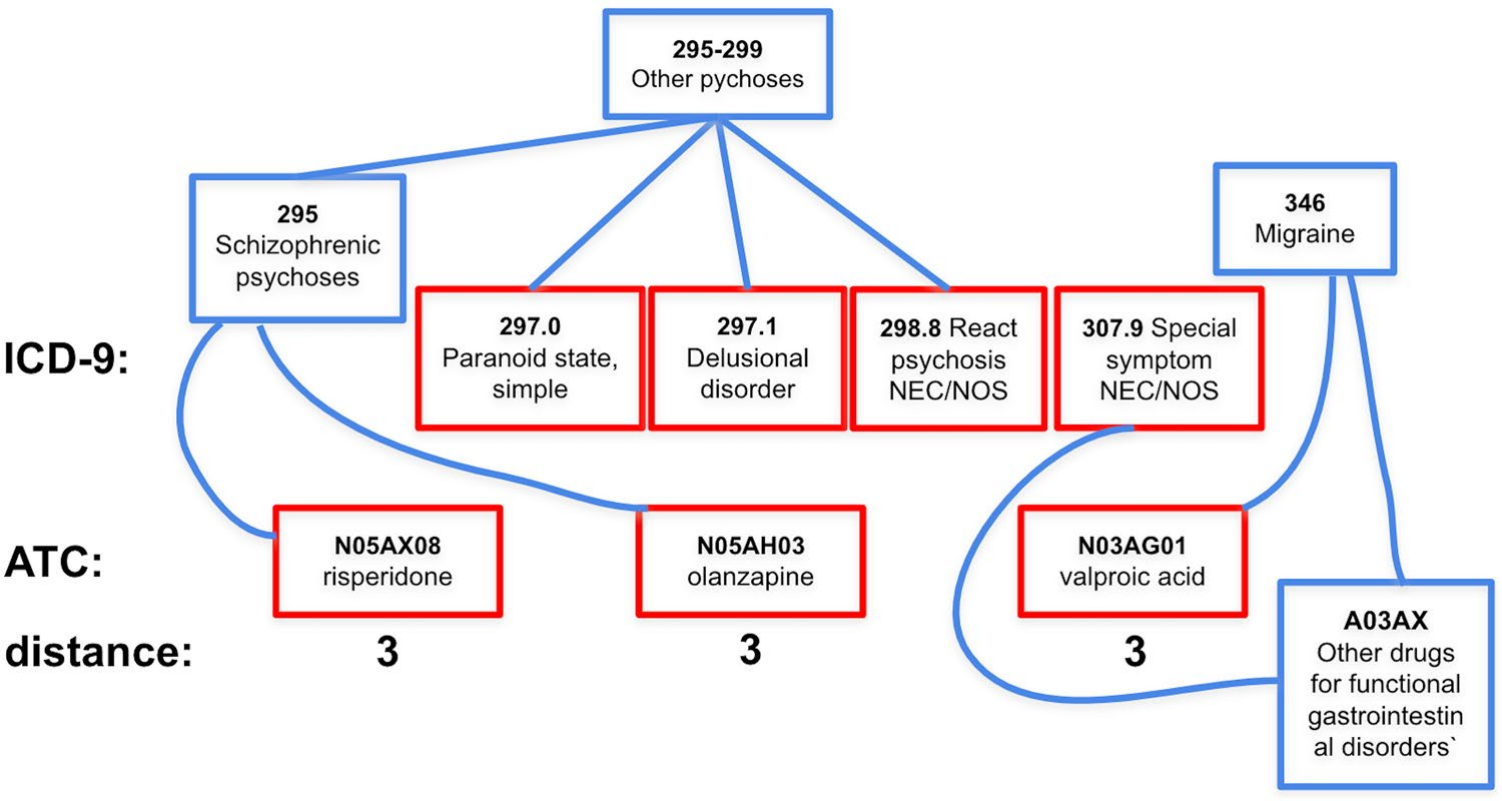
An example of the imputed ATC drugs for a patient. Codes in red frames are observed ICDs and imputed ATCs. The three imputed ATCs are of the same distance to observed ICDs, while their shortest paths may vary.

We then conducted a case study to further ascertain our drug imputation results. For each patient, we measured the distance of each imputed ATC code from the observed ICD codes in the original knowledge graph. We collapsed the last classification level of ICD and ATC for easier analysis, while preserving sufficient granularity. As an example, Fig. 3 shows the top 3 imputed ATC codes (*N05AX08*, *N05AH03*, *N03AG01*) based on the observed ICD codes *297.0*, *297.1*, *298.8*, *307.9* and their parent codes. The distances from an imputed ATC codes to an observed ICD codes are 3 because it requires traversing through other related ICD codes in order to reach to the observed ICD codes. Specifically, the minimal paths of the first two nodes is {*N05AX08*, *N05AH03*} $\rightarrow$ *295* $\rightarrow$ *295-299* $\rightarrow$ *297.0*. The minimal path of *N03AG01* is *N03AG01* $\rightarrow$ *346* $\rightarrow$ *A03AX* $\rightarrow$ *307.9*.

**Figure 4:**
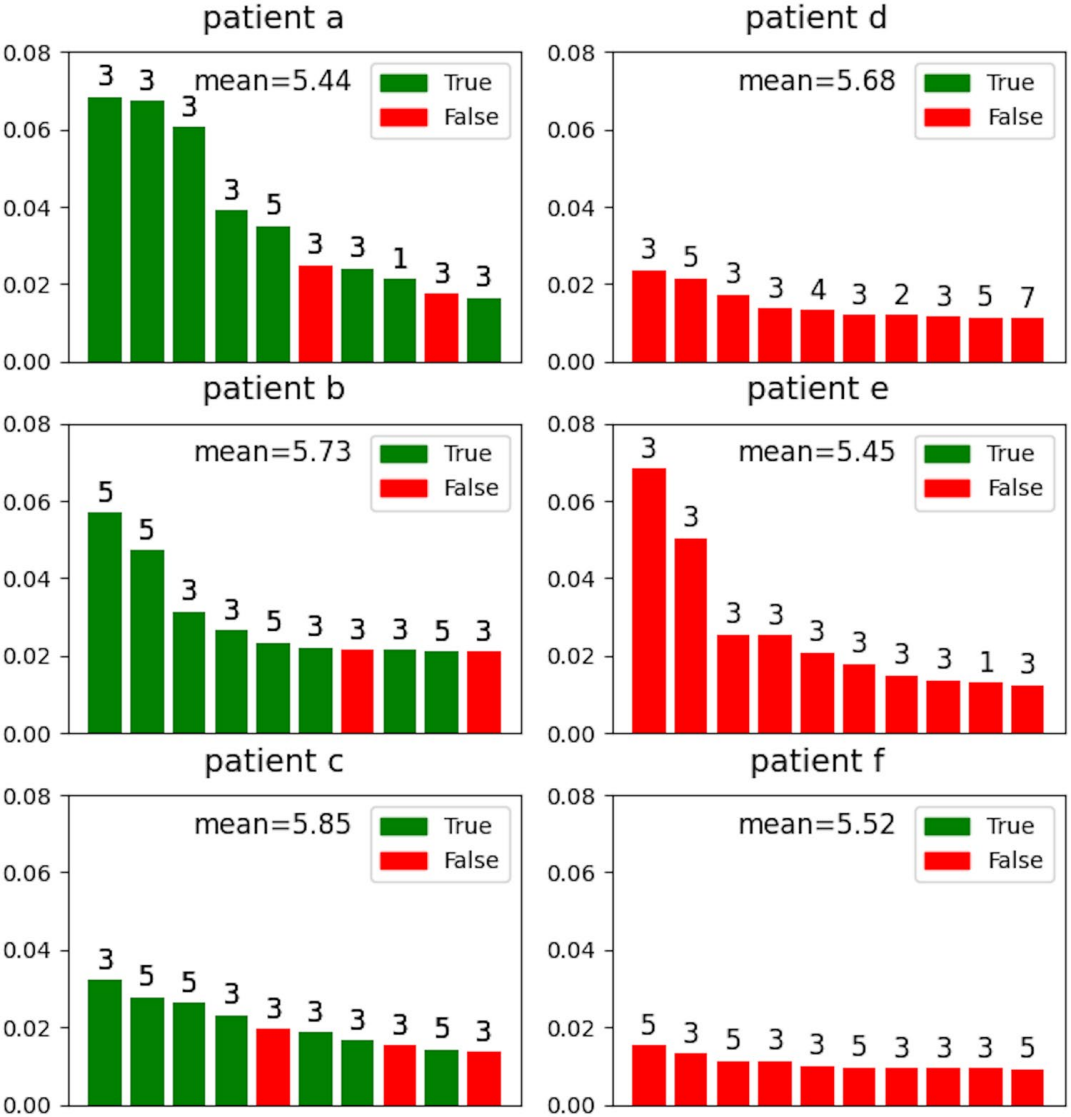
Examples of drug imputation for the 3 most accurately imputed patients and the 3 most inaccurately imputed patients. Each panel displays top 10 imputed drugs of a patient. The height and color of each bar indicates the imputed probability and its correctness, respectively. Annotated above each bar is the shortest distance from each imputed ATC to any of the observed ICD codes for the patient. As a reference, the mean distance indicated in each panel is the average distance from all of the ATC codes to observed ICD codes of the same patient.

**Figure 5:**
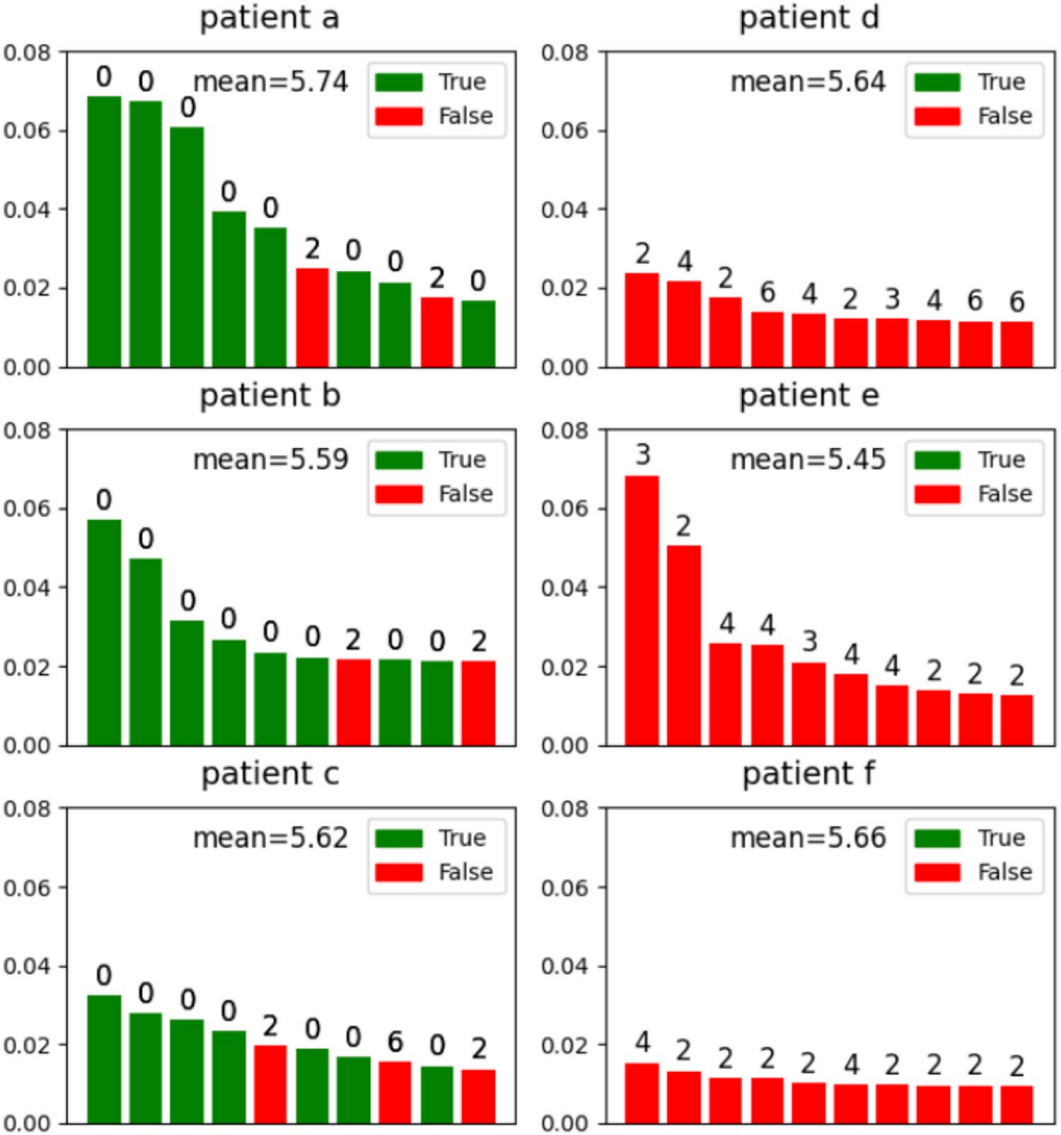
The distance from imputed ATCs to true ATC codes for the 3 most accurately imputed patients and the 3 most inaccurately imputed patients. Similar to Fig. 4, each panel displays the information of the top 10 imputed ATC drugs. The height and color of a bar indicates the imputed probability and whether it is correct. Annotated above each bar is the shortest distance from each imputed ATC to any of the observed ATC codes.As a reference, the mean distance indicated in each panel is the average distance from all of the ATC codes to observed ATC codes of the same patient.

**Figure 6:**
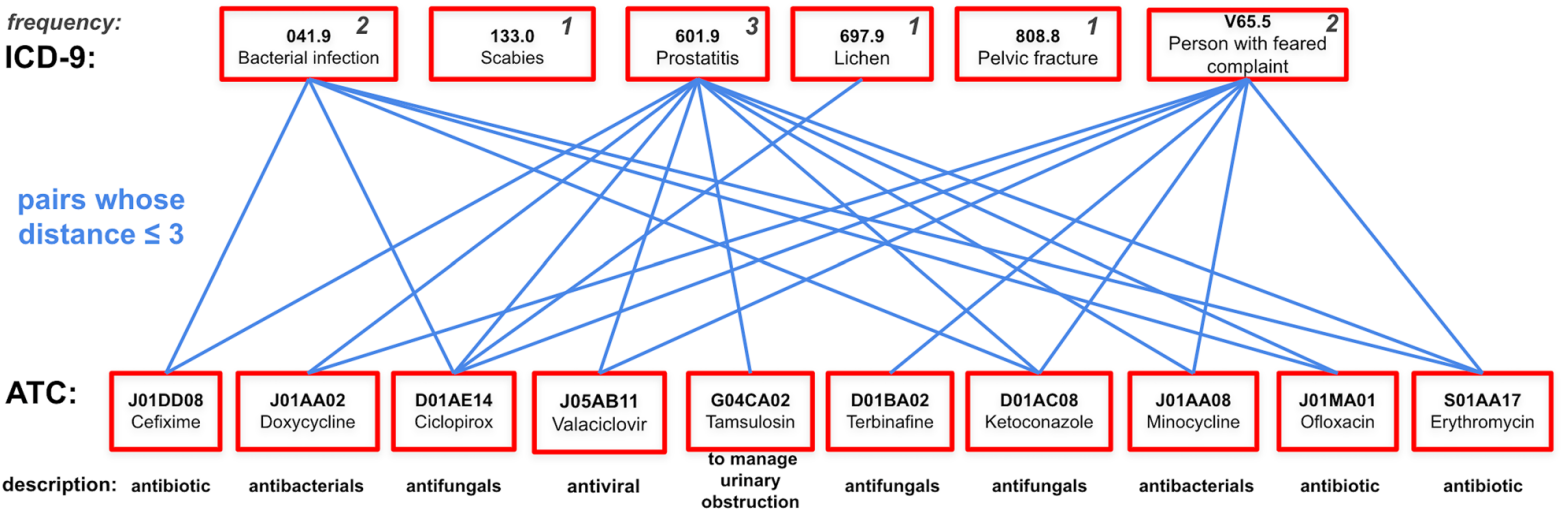
The connection between the observed ICD codes and the imputed ATCs of patient e shown in Fig. 4. ICD-ATC pairs whose distances are no more than 3 are linked. We observed that the imputed ATC codes are closely connected to observed ICD codes. The within-patient frequency for each ICD code is annotated. Short descriptions are provided for each of the imputed ATC codes at the bottom.

Following the above principle, Fig. 4 displays the distance of the top 10 imputed ATC codes from the observed ICD codes for the three most accurately imputed patients (a, b, c) and the three most inaccurately imputed patients (d, e, f). We computed the average distance from all of the ATC codes to the observed ICD codes for comparison. Indeed, compared to the average distance of each patient, all of top 10 imputed ATC codes but one has lower distance from the observed ICD code even for the most inaccurately imputed patients. The only exception is the last drug imputed for patient e, which as distance of 7 from his/her observed ICD codes while average distance is 5.68 in this case. Similarly, most of the top 10 imputed ATC codes are also closer to the observed ATC codes compared to the average distance from all ATC codes to the observed ATC codes for each of the 6 patients (Fig. 5). This means that the recommended ATC codes even for the inaccurately imputed patients are highly related to their observed ICD codes. More concretely, Fig. 6 displays the observed ICD codes and the top 10 recommended ATC codes for patient e (i.e., the second most inaccurately imputed patient). The recommended drugs by GAT-ETM indeed exhibit known associations with the observed disease codes, some of which are observed more than once for the patient (e.g., 601.9 Prostatitis observed 3 times for the same patient).

### GAT-ETM produces meaningful phenotype topics and EHR code embedding

**Figure 7:**
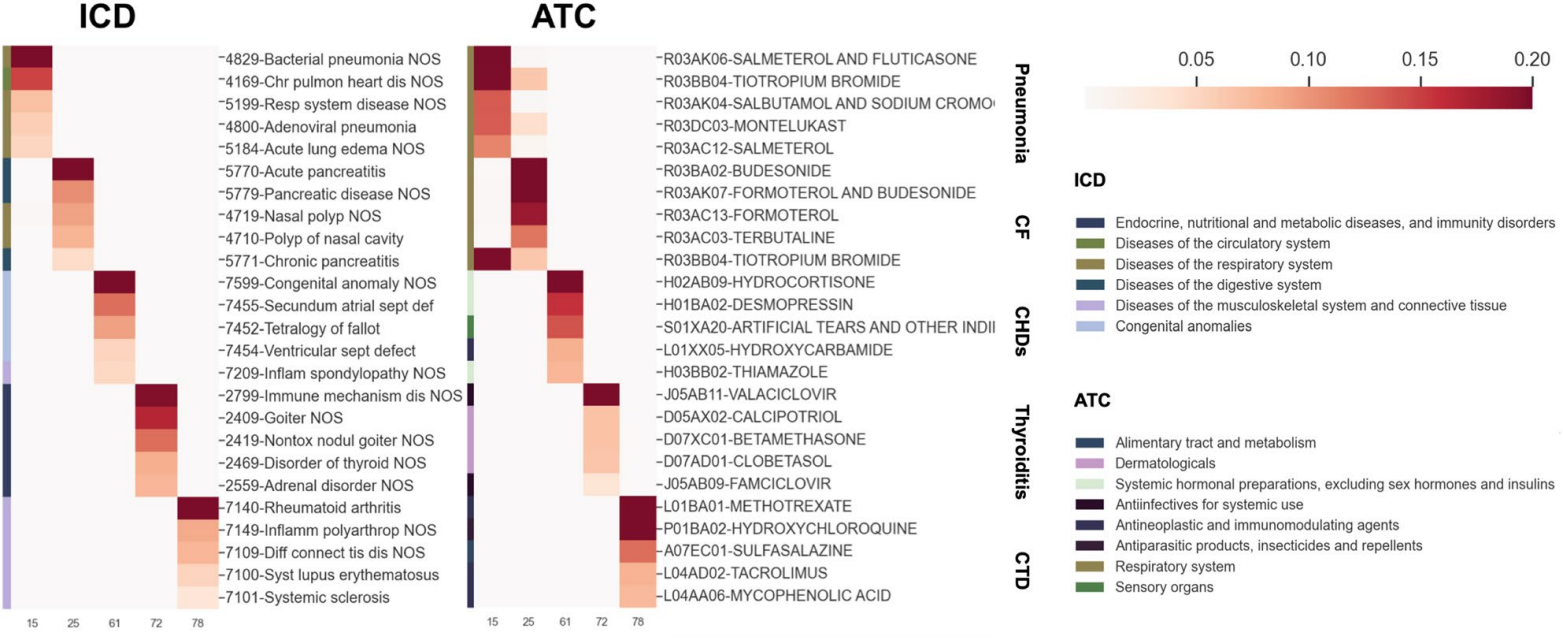
Top 5 EHR codes of the 5 select topics for a diverse set of conditions. The top 5 ICD and ATC codes were displayed for the same topics in the two separate heatmaps. The heatmap intensity is proportional to the probabilities of each code under the topic. The color bar on the left of each heatmap indicates the first-level category of the corresponding code.

To qualitatively assess the disease comorbidity implicated in each topic, we examined 5 randomly chosen topics in terms of their top 5 ICD and ATC codes (Fig. 7). These 5 topics correspond to a set of diverse disease conditions or medications. Indeed, we observe high intra-topic coherence and inter-topic diversity. Specifically, the 5 topics, namely topics 15, 25, 61, 72, and 78, are related to pneumonia, cystic fibrosis (CF), congenital heart defects (CHDs), thyroiditis, and connective tissue diseases (CTD), respectively. Noticeably, CF also causes severe damage to the lung and respiratory system. Hence there is an overlap of the top ATC codes between topics 15 (CF) and 25 (pneumonia). Additionally, many top codes under the same topic are from the same high-level categories or the same subtree of the ICD or ATC hierarchy. The top codes that are not in the same categories are also clinically relevant. For example, topic 25 cystic fibrosis triggers both lung diseases and respiratory diseases.

**Figure 8:**
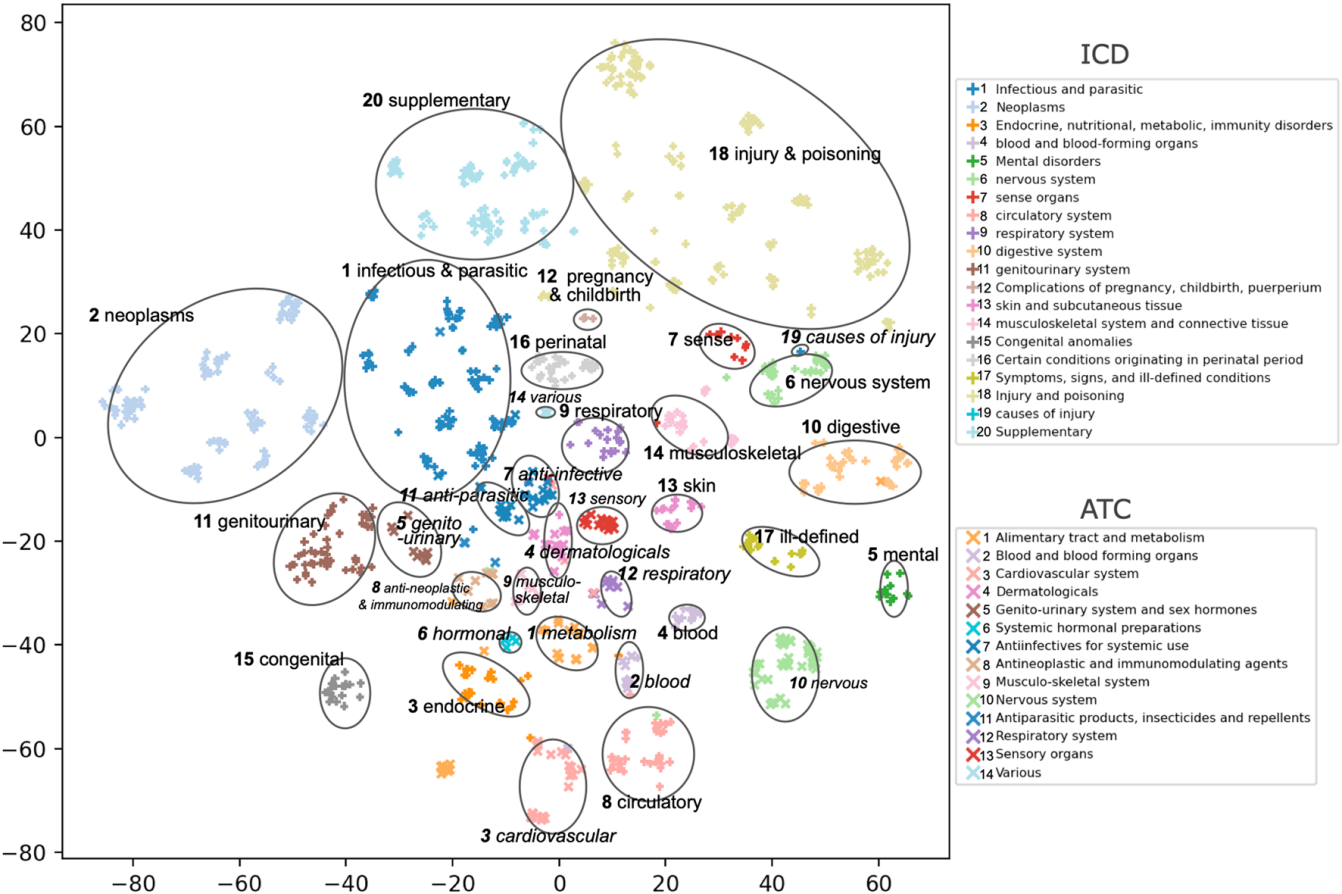
Clustering of EHR codes based on their learned embedding $\varvec{\uprho }$ by our GAT-ETM. t-SNE was applied to the embedding to reduce their dimensions from *L* to 2 to allow visualization of the code clustering. As shown in the legend, shape $+$ and $\times$ indicate ICD and ATC code, respectively; colors indicate different high-level categories. Aligned ICD and ATC categories are assigned identical or similar colors. Within ICD/ATC vocabularies, nodes of the same category are grouped together. Each group was circled and labeled with abbreviations. ICD and ATC group names are shown in regular and italic fonts, respectively.

We then visualized the code embedding of both ICD and ATC using t-distributed Stochastic Neighbourhood Embedding (t-SNE) (Fig. 8). As a proof-of-concept, the codes do not only cluster into similar categories but also cluster close to each other if they exhibit putative therapeutic relations. For example, ICD codes in “13-Skin and subcutaneous tissue" category and ATC codes in “4-Dermatologicals" category (in pink color) cluster closely together; ICD codes in “3-endocrine, nutritional and metabolic diseases, and immunity disorders" category and ATC codes in “1-Alimentary tract and metabolism" (in orange color) cluster together; ICD codes in “1-infectious and parasitic" and ATC codes “7-antiinfectives for systemic use" and “11-antiparasitic products, insecticides and repelients" cluster together; ICD codes in “8-circulatory" category cluster together with ATC codes in “3-cardiovascular system".

## Discussion

In this study, we present an end-to-end graph-embedded topic model that: (1) learns interpretable topic and code embeddings in the same embedding space; (2) is able to handle multimodal data; and, (3) leverages a medical knowledge graph to improve performance quantitatively and qualitatively. We compared the performance of GAT-ETM against several existing methods on the EHR reconstruction task, automated phenotyping task and drug imputation task. GAT-ETM consistently outperformed the alternative methods in these tasks. These results showcase the benefits of our end-to-end learning framework. Additionally, we show that integrating knowledge graphs of multiple views (i.e., ICD and ATC in our context) brings complementary information to characterize the same phenotypes. Moreover, our graph augmentation strategy improves the information flow through the taxonomy graphs. Qualitative analysis further illustrated that GAT-ETM learned coherent phenotype topics and meaningful latent embedding of the EHR codes.

In future work, we will explore four promising directions. First, we will leverage large and comprehensive biomedical knowledge graphs with richer relations that comprise not only ICD codes and ATC codes but also other codes such as gene ontology terms available from Universal Medical Language System (UMLS) and elsewhere. Additionally, we will extend GAT to multi-relational graphs to account for heterogeneous graphs. For example, a drug may treat or induce a disease, which should be considered as different types of relations. Furthermore, in this work, for drug graph we use ATC code that merely has drug classification hierarchy information. We plan to incorporate drug-drug interactions (DDI) in future multi-relational graph-based approaches so that we can impute drugs without adverse effects.

Second, topic identifiability is a challenge in completely unsupervised topic modeling. Guided topic models^40,41^ make use of expert-curated phenotype concepts such as PheCodes and Clinical Classification Software (CCS)^42^ to guide disease topic inference. In the future, we will incorporate the guided mechanism as anchor topic nodes in graph embedding learning to generate identifiable and presumably more interpretable topics.

Third, attention mechanism enables us to track the contribution of input features^22,43^. GAT-ETM utilizes a GAT network, where each node computes attention weights over its neighbors and then controls information flow through the attention weights. This provides venues to look into the blackbox of the deep learning framework to understand the disease connections. We will find effective ways to dissect the attention weights among EHR codes in order to predict their comorbidity associations.

Lastly, we will harness longitudinal EHR data. We will extend our model to a dynamic topic model^44^ that accounts for the evolution of patients’ health status over time. There are several ways to track patients’ health status. One is to regard longitudinal visits as document series with timestamps. Based on this, we can infer disease progression and train predictive models. We will also need to consider irregular visits in outpatient data when modelling longitudinal EHR. Another approach is to group visits by fixed partitions, e.g. age. Such approach can model progression of age-dependent diseases such as hypertension.

## Data Availability

The data generated and analyzed during the current study are not openly available due to privacy laws and policies in Quebec Canada. The GAT-ETM code is publicly available at https://github.com/li-lab-mcgill/GAT-ETM.
